# Viability and functional impact of probiotic and starter cultures in salami-type fermented meat products

**DOI:** 10.3389/fchem.2024.1507370

**Published:** 2024-11-26

**Authors:** Emma Mani-López, Ricardo H. Hernández-Figueroa, Aurelio López-Malo, Jocksan I. Morales-Camacho

**Affiliations:** Chemical, Food and Environmental Engineering Department, Universidad de las Américas Puebla, Cholula, Mexico

**Keywords:** meat fermentation, physicochemical properties, meat starter, meat probiotic, mixed culture

## Abstract

Salami, a well-known fermented meat product, is made from selected ground meat mixed with curing agents and spices. This work aimed to determine the viability of *Lactiplantibacillus plantarum* (as a starter), *Lactobacillus acidophilus* (probiotic microorganism), and their mixture during the fermentation and ripening of a salami-type product, evaluate the microbiological and physicochemical changes and assess the sensory acceptability of the final product. *L. acidophilus* has not been sufficiently explored as a probiotic in fermented meats, especially in terms of its effects on fermentation and sensory qualities. Salami-type products were formulated and fermented for 48 h at 32°C, and then ripening took place at 8°C for 13 days. pH, titratable acidity, *Lactobacillus* counts, and contaminating microbiota were analyzed during the process. Sensory evaluation was analyzed in the final products. The salami-type formulation served as an effective medium for growing microorganisms, with the populations of starter and probiotic cultures exceeding 10^8^ CFU/g after fermentation and ripening for 15 days. The pH of the end products was ∼5.1, titratable acidity ∼2.5%, and aw ∼0.83. During fermentation and ripening, a significant reduction in total mesophilic aerobic bacteria (>7 logs), coliforms, and *Staphylococcus aureus* (>8-fold reductions) were observed. The sensory evaluation results indicate that the product’s attributes are not influenced by the type of bacteria used, as no significant difference was found (*p* > 0.05). The results show that *L. acidophilus*, *Lactiplantibacillus plantarum*, or their mixture can be used as a starter culture in fermented meat products. Using *L. acidophilus*, whether alone or in combination, is a viable option that preserves the characteristics of the fermented product and may enhance the benefits of probiotic consumption.

## 1 Introduction

Fermented foods, used for thousands of years, might promote consumer health since they may contain beneficial microbes, mainly lactic acid bacteria (LAB), responsible for texture, flavor, and preservation (Ouwehand and Röytiö, 2015; Hernández-Figueroa et al., 2024). The fermentation of meat dates back centuries, when its native microbiota naturally fermented meat cuts without controlled processing conditions to preserve meat (Munekata et al., 2020). One of the main fermented meat products is salami, with a hard consistency, and is highly seasoned. It is prepared from selected ground meat, mixed with curing agents and spices, brought to low temperatures, stuffed, dried with air or smoke at elevated temperatures, and controlled humidity conditions. The lactic acid production occur during salami’s fermentation, providing the characteristic flavor and color (Kumar et al., 2017; Wang D. et al., 2022; Ashaolu et al., 2023).

The use of starter cultures offers several advantages over spontaneous fermentation, such as better control of the fermentation process, shorter ripening time, reduced risk of pathogenic microorganism growth, and improved consistency in product quality between batches. However, choosing the proper starter culture for a functional meat product is challenging due to the complexity of each step and the numerous required assays (Munekata et al., 2020). LAB from the genera *Lactobacillus*, *Pediococcus*, *Leuconostoc*, *Lactococcus*, and *Enterococcus* are typically used as starters in fermented meat products. These bacteria help lower the pH of the meat product by producing lactic and acetic acids through the glycolysis pathway (Rocchetti et al., 2021). In consequence, the meat’s color enhances, inhibits pathogenic bacteria, and contributes to the characteristic flavor of cured meats. The production of bioactive peptides by selected LAB (including *Lactobacillus plantarum* and *Lactobacillus acidophilus*) during the fermentation and ripening can significantly enhance the nutritional and functional value of the product. Some examples of traditional fermented meat products, the type of meat used, and the starter cultures commonly employed are: Italian salami (from pork or beef) is a classic example of a dry-cured sausage where LAB (*Lactobacillus plantarum* or *L. acidophilus*, along with *Pediococcus*, *Leuconostoc*, *Lactococcus*, and *Enterococcus*) are introduced to ensure acidification, promoting texture and flavor development (Fraqueza et al., 2016; Laranjo et al., 2019). *Fuet* is a traditional Catalonian (Spain) pork dry-cured sausage fermented using LAB to achieve the desired pH and safety using *Lactobacillus sakei* as a starter culture (Ferrer-Bustins et al., 2023). *Chorizo* (from Spain) is a fermented (*Lactobacillus curvatus* as a starter) paprika-flavored pork sausage (Caro et al., 2022). *Sucuk* (from Turkey) is a spicy, fermented beef or lamb sausage typically using starter culture *Lactobacillus plantarum* (Kamiloğlu, 2022; Kaban et al., 2022).

Probiotics are live microorganisms that confer health benefits to the host when administered in adequate amounts (FAO/WHOFood and Agriculture Organization of the United Nations/World Health Organization, 2002). In the context of fermented foods, these beneficial bacteria can enhance gut health, modulate immune responses, and contribute to the prevention of various diseases (World Gastroenterology Organization Global Guidelines, 2017). Probiotic foods are considered functional foods due to providing health benefits beyond traditional nutrients. The global market of functional foods is expanding at a compound annual growth rate of 8.5% from 2022 to 2030 due to functional food health benefits and consumers have begun to pay attention on their lifestyles and healthy diets (Grand View Research, 2024a). In addition, fermented meat is ranked as a growing sub-segment probiotic product from 2018–2030 in the category of probiotic food and beverages (Fairfield, 2023; Grand View Research, 2024b). Probiotics are traditionally combined with starter cultures in fermented dairy products such as yogurt and milk. They have also been found in other types of fermented foods, such as soy products, grain-based foods, vegetable or fruit foods, and meat products, among many others (Zhao et al., 2019). Traditionally, non-probiotic lactobacilli strains are used as starter cultures; however, some probiotic LAB strains can be used during the fermentation of meat products since they produce acid and meet the characteristics to be considered probiotics (Pennacchia et al., 2004; Pennacchia et al., 2006; Coelho et al., 2019; Kumar et al., 2017; Ammor and Mayo, 2007). Arihara et al. (2008) supplemented *Lactobacillus gasseri* JCM1131 in pork sausages as a probiotic microorganism to improve product safety. Probiotic strains like *L. acidophilus*, *L. casei*, and *Lactobacillus rhamnosus* can be used alongside traditional LAB to maintain fermentation quality and offer health-promoting properties. These LAB assist in acidification while providing potential probiotic benefits. Few studies are focused on investigating the health benefits of probiotic meats consume. The intake of probiotic salami (daily 30–50 g) with *L. acidophilus* LAFTI^®^ L10, *L. rhamnosus* HN001, or *Lactobacillus paracasei* LTH 2579 reduced the *Listeria* fecal levels, decreased markers of inflammatory processes (C-reactive protein and tumor necrosis factor-α), or stimulated immunological response, respectively (Mahoney and Henriksson, 2003; Pérez-Burillo et al., 2020; Jahreis et al., 2002; Manassi et al., 2022). Thus, exist evidence of health benefits from fermented probiotic meats intake. The synergistic use of probiotics and starter cultures in fermented meat production could yield a safe, nutritious, and highly palatable product (Centeno and Carballo, 2014). *L. acidophilus* has been scarcely investigated as probiotic in salami manufacture and its impact in fermentation and sensory attributes, since other probiotic *Lactobacillus* species are preferred. The probiotic supplementation in salami-type requires the assessing of the viability of microorganisms during fermentation and ripening to ensure the health benefits of probiotics and the effectiveness of starter cultures, particularly for maintaining the functional properties of the final product. Evaluation of microbiological, physicochemical, and sensory characteristics is essential for developing high-quality salami-type. Including sensory evaluation offers valuable consumer insights often overlooked in technical studies, ensuring the final product is safe, nutritious, and appealing. Therefore, this work aimed to determine the viability of *Lactiplantibacillus plantarum* (as a starter), *L. acidophilus* (probiotic microorganism), and their mixture during the fermentation and ripening of a salami-type product, evaluate the microbiological and physicochemical changes that occur during fermentation and ripening and assess the sensory acceptability of the final product.

## 2 Materials and methods

### 2.1 Materials and culture conditions

The meat was purchased from a supermarket in individual pieces and subsequently frozen at −18°C for 24 h. Similarly, the fat (lard) was obtained from a butcher shop and subjected to the same freezing conditions. Curing salt, ground black pepper, sodium erythorbate, and minced garlic were purchased from McCormick Pesa SA de CV (Cuautitlan, Estado de Mexico, Mexico), and salt and sugar were acquired from a local supermarket (Puebla, Puebla, Mexico).

The starter culture *Lactobacillus plantarum* (new name *Lactiplantibacillus plantarum*) (BioCarna^®^ Protect ALC) was acquired in lyophilized form from Danisco Culture. *Lactobacillus acidophilus* LA-5 was obtained from Chr. Hansen. Both cultures were suspended in de Man Rogosa Sharpe (MRS) broth (Difco, BD, Sparks, MD). Both bacteria were incubated at 35°C ± 1.0°C for 48 h (10^9^ CFU/mL), and cells were harvested by centrifugation at 8,000 *g* for 10 min at 4°C (Marathon 21K/R, Fisher Scientific, Germany), and washed twice with phosphate buffer (pH 7.0). The wet cell pellets were weighed and refrigerated to use on the same day in the salami-type sausage.

### 2.2 Salami-type preparation

The ingredients used to prepare the salami-type meat product are presented in Table 1. Utilizing a cutter, the meat was cut into pieces 1–2 cm, while the fat was cut into pieces ranging from 2 to 3 mm. Subsequently, the meat was combined with the fat within the cutter, and all dried ingredients were carefully added. Upon the mixture was completed, a starter culture, the probiotic culture, or the mixture of both bacteria was introduced for salami-type control or probiotic (for the mixture, the starter culture was incorporated initially, followed by the addition of the probiotic). The quantity of the starter, probiotic, or mixture added (Table 1) was based on the findings of prior experiments and the dilution of viable cell counts in the pellet (10^9^ CFU/g) in the salami-type product formulation (Table 1). This indicates that the initial count is expected to exceed 10^5^ CFU/g, which is adequate to ensure a sufficient number of bacteria to initiate the fermentation process effectively.

**TABLE 1:** Salami-type sausages formulation.

Ingredient	%
Lean pork	68.56
Lard	29.38
Salt	1
Curing salt	0.3
Sugar	0.5
Sodium erythorbate	0.05
Ground black pepper	0.01
Minced garlic	0.2
Starter (*Lactiplantibacillus planatrum*)	0.1 g (10^9^ CFU/g)
Probiotic (*Lactobacillus acidophilus*)	0.1 g (10^9^ CFU/g)

The mix was then stuffed in cellulose cases (diameter 50 mm) and separated into pieces of 15 cm (approximately 150 g) for each treatment. For the fermentation step, salami-type sausages were placed in an environmental incubator (Line Benchtop Environmental Chamber, Melrose, Fl) at 32°C for 48 h, and relative humidity (RH) remained between 80% and 90%. After this time, the ripening process was carried out. For this, the sausages were kept in the same incubator, but the conditions were changed to 8°C (refrigeration), and the relative humidity level was reduced (40%). The sausages were kept in these conditions for 13 days (when the pH of the sausages was between 5.1 and 5.2).

### 2.3 Starter and probiotic analysis

The starter culture and probiotic bacteria (in duplicate) were counted immediately after preparation and during fermentation (after 24 h) and ripening (4–13 days) of salami-type. Ten grams of salami-type was weighed and suspended in 90 mL of peptone water and homogenized in a stomacher 80 lab blender (Seward Ltd., West Sussex, England). Ten-fold serial dilutions were made in sterilized peptone water and inoculated in MRS agar (Difco, BD, Sparks, MD). Petri dishes with *L. acidophilus* and the starter culture were incubated at 37°C for 72 h anaerobically.

### 2.4 Microbiological analysis

The microbiological quality and safety of cured/aged meat products like salami is crucial and involves monitoring specific pathogenic microorganisms and quality indicators throughout production. Microbial counts were performed for each indicator bacteria. Adequate decimal dilutions were prepared into peptone water, and 1 mL was spread on Baird-Parker agar (Merck KGaA, Darmstadt, Germany) for *Staphylococcus aureus,* plates were incubated at 35°C for 48 h (FDA, 2019); XLD agar (Merck KGaA, Darmstadt, Germany) for *Salmonella*, the inoculated plates were incubated at 37°C for 24 h (Arrioja-Bretón et al., 2020); Oxford agar plates (Difco, BD, Sparks, MD) for *Listeria monocytogenes* were incubated at 37°C for 24 h (Barbosa et al., 2015) and MacConkey agar (Merck KGaA, Darmstadt, Germany) for *E. coli* was incubated for 18 h at 37°C (Kyere et al., 2015). In addition, total mesophilic aerobic bacteria (TMAB) were counted using standard methods agar (Bioxon, BD, Edo. de Mexico, Mexico) (NOM-092-SSA1-1994, 1995); inoculated plates were incubated for 24 h at 37°C. For total coliform counts, violet-red bile agar (Bioxon, BD, Edo. de Mexico, Mexico) was utilized, and plates were incubated at 37°C for 24 h (NOM-113-SSA1-1994, 1995). Yeasts and molds were cultivated in potato-dextrose agar (Bioxon, BD, Edo. de Mexico, Mexico) and plates incubated at 25°C for 72 h (NOM-111-SSA1-1994, 1995). The detection limit for *Staph. aureus, Salmonella, L. monocytogenes, Escherichia coli,* and microbial groups counts is 10 CFU/g.

### 2.5 Physicochemical and proximal analysis

The salami-type sausage’s moisture, fat, and protein content were determined as follows: moisture content was determined using 5 g of sample by weighing the difference in an oven (Felisa, México) at 105°C ± 2°C. Proteins were quantified following the Micro-Kjeldahl method using a digestor unit (Labconco, Model 23,080, USA) and a Nitrogen/Protein Distiller (Labconco, Model: Rapidstill II, USA), 0.2 g of each salami-type and a factor conversion of 6.25. Fat was determined in 5 g of salami-type by Soxhlet extraction (E−500 Büchi, Flawil, Switzerland) with petroleum ether for 6 h (the sausage was dried previously at 60°C for 4 h).

The pH was measured by electrode immersion following the method 32.016 AOAC (1996) using a potentiometer (UB-10, Denver Instrument, Bohemia, NY) after preparation and during fermentation and ripening of salami-type. Titratable acidity was determined by titration with 0.1 N NaOH and expressed as a percentage of lactic acid. Water activity (a_w_) was measured utilizing an Aqualab Serie 3 TE (Decagon Device Inc., Pullman, WA).

### 2.6 Sensory evaluation

Sensory quality and acceptability of salami-type sausages were assessed using an affective sensory test with a nine-point hedonic scale, where one corresponds to “I do not like it very much” and nine to “I like it very much”; an average of ∼6 was considered acceptable. The assessment was performed by a panel of 25 untrained judges, who were representative of typical consumers of fermented meat products, selected to reflect the demographic profile of consumer preferences. Although the panelists were untrained in professional sensory analysis, an orientation session was provided before the assessment to familiarize them with the evaluation process and ensure consistency. During this session, they were instructed to rate attributes such as taste, odor, color, texture, and overall acceptability to minimize variability in their responses. The consumer panel size of 25 judges was selected based on the practical limitations of the study, balancing resources, and the need for meaningful results. Given this panel size, the minimal detectable difference on a nine-point hedonic scale is approximately one point, sufficient for identifying moderate differences in sensory attributes between samples (Hough et al., 2006). The chosen panel size is appropriate for evaluating noticeable variations in product acceptability, ensuring a reliable assessment within the scope of the study.

The Research and Ethics Committee on Sensory Evaluation of Foods of the doctoral program in Food Science of the Universidad de las Américas Puebla approved the protocol for the sensory evaluation carried out in this work on 16 November 2023 (document number SEDCL-2023/019). Sensory panel. The panelists were informed about the sausages (control, using a starter and probiotic) and the ingredients used. All participants signed informed consent by affirmative replay the following statement: “I am aware that my responses are confidential, and I agree to participate in this sensory evaluation”. They were also informed they could withdraw from the test without a reason; we explicitly stated, “The products are safe to assess the visual color, odor, and flavor inspection” and that participants’ data would not be disclosed without their knowledge.

### 2.7 Statistical analysis

The viability of starter and probiotic counts, pH, titratable acidity, and data obtained from the sensory evaluation were analyzed using the Minitab 20 software, employing a one-way ANOVA and Tukey’s mean comparison (*p* < 0.05). In addition, the statistical analysis involved two-sample t-tests to assess differences in pH and titratable acidity (%) changes using *L. plantarum, L. acidophilus*, and a mixed culture of both microorganisms. The t-tests were applied to compare mean values between pairs of treatments. For each test, equal variances were assumed to assess the statistical significance of the variations observed. This approach allowed us to evaluate whether the distinct microbial treatments yielded significant differences in acidification trends over the fermentation and maturation periods.

## 3 Results and discussion

### 3.1 Salami-type sausages moisture, fat, protein content, and a_w_

The moisture, fat, and protein content changes in the salami-type sausage across different processing stages (Table 2) are consistent with trends reported in the literature. During fermentation, water loss occurs (∼2%) due to elevated temperature and humidity. The fat and protein content undergoes minimal changes as fermentation mainly affects moisture levels (Table 2). Throughout ripening and drying, moisture decreases significantly (∼20%) while protein and fat become more concentrated (∼9% and 3%, respectively) due to water loss. By day 5, moisture content decreases by 10%–15%, and fat and protein content increases in concentration (∼5% and 2%, respectively). By day 10, the drying process further reduces moisture content (∼10%) (Table 2), and the product typically reaches a pH of 5.1–5.2, indicating readiness for consumption. Likewise, Roseiro et al. (2010) observed significant moisture loss and concurrent increases in fat and protein percentages due to dehydration while drying in Portuguese traditional dry-fermented sausages. Another study showed moisture content reductions while increasing fat and protein percentages as water loss concentrates these macronutrients in the product (Spaziani et al., 2009). The observed trends in the data align well with these studies, supporting typical composition changes during sausage drying and ripening processes.

**TABLE 2:** Moisture, fat, and protein content of fresh salami-type sausage inoculated with *Lactiplantibacillus plantarum* and *Lactobacillus acidophilus* (mixture) during the fermentation and ripening process.

Stage	Moisture (%)	Fat (%)	Protein (%)
Initial (pre-fermentation)	48.07 ± 0.57	36.30 ± 0.51	13.73 ± 0.15
End of fermentation	46.02 ± 0.65	37.04 ± 0.42	14.01 ± 0.13
Day 5 of ripening	36.10 ± 0.81	41.25 ± 0.54	15.60 ± 0.16
Day 10 of ripening	26.09 ± 0.47	46.54 ± 0.36	17.60 ± 0.17

The a_w_ of the salami-type sausages at the end of the ripening stage was 0.844 ± 0.010, 0.832 ± 0.015, and 0.835 ± 0.08 for *L. plantarum*, *L. acidophilus*, and *L. plantarum* plus *L. acidophilus* mixture, respectively. A wide range of a_w_ were reported for commercial salamis between 0.65 and 0.97 (Lee and Styliadis, 1996). In another study, Sicilian salami recorded a_w_ values of 0.81–0.83 (Moretti et al., 2004). Thus, the probiotic salami-type a_w_ is in the range values of commercial salamis and previous reported for Sicilian one. There are no specific values of salami’s a_w,_ and different criteria are suggested to guarantee product safety without unnecessarily rejecting products. Recommended a_w_ values range between 0.85 and 0.92 to prevent microbial growth or trichinae larvae from surviving (Lee and Styliadis, 1996). Thus, the a_w_ of the present salami-type sausages were under these values, it guarantees the safety of the product since no bacteria or parasite can grow.

### 3.2 pH and titratable acidity changes during fermentation and ripening

**FIGURE 1:**
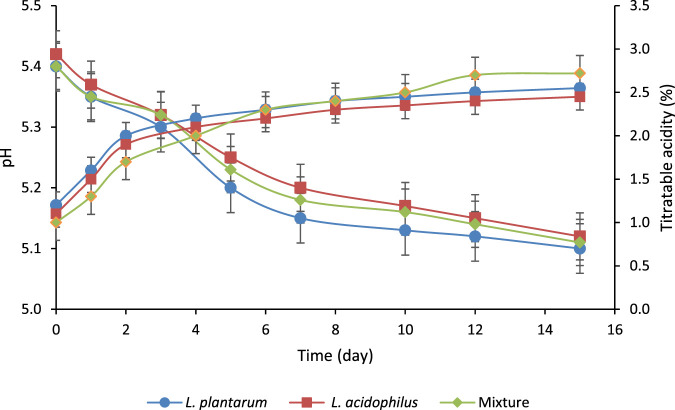
pH and titratable acidity (%, lactic acid) during fermentation and ripening of salami-type sausages fermented with *Lactiplantibacillus plantarum*, *Lactobacillus acidophilus* or a mixture of both.

Figure 1 shows the pH and titratable acidity during the fermentation and ripening of salami-type sausages. The pH of all samples decreased from approximately 5.4 at day zero to around 5.1–5.2 by day 15, with the most substantial reductions occurring within the first 5 days. Varnam and Sutherland (1995) reported that salami ripening ends when the product reaches a pH of 5.1–5.2; thus, a correct pH was achieved at the end of ripening. The sausage inoculated with *L. plantarum,* the pH decreases slightly more rapidly than inoculated with *L. acidophilus*, or with the LAB mixture. However, there is no significant difference (*p* > 0.05) between the values of the three sausages, reaching the pH values proposed for their ripening. The sausages prepared with *L. plantarum,* and the mixture of microorganisms follow the same trend. In contrast, the sausage prepared with *L. acidophilus* follows a linear trend (Figure 1). The pH and acidity changes during the fermentation of the salami-type products using single cultures of *L. plantarum* and *L. acidophilus*, as well as a mixed culture, were analyzed with Two-sample t-tests. The results showed no significant differences in pH or acidity among the treatments, with *p*-values for pH of 0.588, 0.759, and 0.804 and *p*-values for acidity of 0.650, 0.805, and 0.892, respectively. These findings indicate that neither single nor mixed cultures significantly impacted acidification, suggesting comparable acidification kinetics across the treatments. Thus, the mixed culture does not enhance acidification performance over individual cultures. The study’s findings on pH changes in salami-type products align with the well-established principles of meat fermentation, where LAB plays a pivotal role in lowering pH by producing lactic acid through carbohydrate metabolism. The values obtained in this research (5.1–5.2) align with those reported by Stangierski et al. (2023), who observed pH values of 5.15 in salamis after 15 days of fermentation. Similarly, Dasiewicz et al. (2024) reported values of 4.97 for salami-type sausages after 21 days containing *Lactobacillus sakei* and *Staphylococcus carnosus*.

Lactic acid is the primary acid LAB produce as starters and/or probiotic cultures. In Figure 1, the increase in titratable acidity (lactic acid) is almost 100% in the three cases after 15 days. Despite no significant differences (*p* > 0.05) in titratable acidity were observed during the fermentation and ripening, the sausage with the highest lactic acid was prepared with *L. plantarum,* while the sausage made with *L. acidophilus* had the lowest production of lactic acid. The rapid acidification when *L. plantarum* is used as culture was expected because it is known for robust acid production, particularly in meat matrices, where it is highly adapted to thrive in high-salt and low-oxygen environments (Sirini et al., 2023). *L. acidophilus* produces lactic acid at a slower rate, as reflected in the more gradual acidity increases in the sausages inoculated solely with this strain. Unexpected slow acid production was observed in the sausages inoculated with the mixed cultures since a synergistic behavior should be observed. However, competition for nutrients may occur during the fermentation and ripening stages. Polizzi et al. (2024) observed that a starter culture containing *S. carnosus*, *Staphylococcus xylosus*, and *L. sakei* led to faster acidification in “Salame Napoli” (an Italian dry-cured sausage) than the sausage fermented with *S. xylosus* and *L. plantarum.*

In fermented meat products, reducing pH plays a vital role in inhibiting the growth of pathogenic and spoilage microorganisms, thereby extending shelf life. As shown in Table 3; Figure 1, the pH decreases rapidly during the initial days of fermentation, reaching levels that inhibit pathogens such as *Salmonella* spp., *L. monocytogenes*, *Staph. aureus*, and *E. coli*, with undetectable or significantly reduced counts by day 5–6. This reduction in pH and increased titratable acidity creates an environment unfavorable to pathogens, enhancing product safety. Furthermore, it is possible that the starter cultures (*L. plantarum* and *L. acidophilus*) could produce bacteriocins or bioactive peptides with antimicrobial properties. These compounds would provide an additional barrier against pathogens, complementing the inhibitory effects of low pH and acidity. Bacteriocins and bioactive peptides are known for their ability to target specific bacteria, potentially offering targeted antimicrobial action that could further reduce the risk of contamination and contribute to the stability and safety of fermented meat products (Munekata et al., 2020). Studies by Leroy and De Vuyst (2004) highlight that higher lactic acid levels in fermented sausages improve microbial safety by inhibiting undesirable bacteria, such as *L. monocytogenes* and *E. coli*.

**TABLE 3:** Microbial counts (CFU/g) of contaminated microbiota in the salami-type sausages inoculated with *Lactiplantibacillus plantarum* and *Lactobacillus acidophilus*.

Microbial group	Initial counts (day 1)	Fermentation (day 2)	Ripening (day 5–6)	Ripening (day 10–15)
*Salmonella* spp.	<10	<10	<10	<10
*Listeria monocytogenes*	<10	<10	<10	<10
*Staphylococcus aureus*	80	10	<10	<10
*Escherichia coli*	130	10	<10	<10
Total coliforms	160	10	<10	<10
Yeast and molds	100	10	10	<10
Total mesophilic aerobic bacteria (TMAB)	280,000	3,500	680	370

### 3.3 Lactic acid bacteria viability during fermentation and ripening of salami-type sausages

**FIGURE 2:**
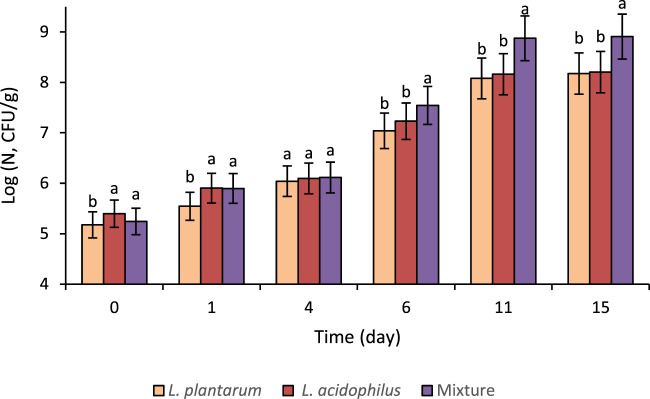
Microbial viability in the salami-type meat product of inoculated starter culture (*Lactiplantibacillus plantarum*), probiotic (*Lactobacillus acidophilus*), and the mixture of both during fermentation and ripening.

The LAB count analysis was conducted immediately after the salami-type products were prepared, during fermentation and ripening. Figure 2 shows the viability of *L. plantarum* (starter culture), *L. acidophilus* (probiotic), and their mixture inoculated in the salami-type fermented meat product. Figure 2 shows that *Lactobacillus* grew well in all cases. The recommended level of probiotics depends on the microorganism type and the health benefit; thus, variable amounts of probiotics are required. The food industry and the US FDA recommended that the minimum probiotic food must have at least 10^6^ CFU/g (Tripathi and Giri, 2014), and the product obtained in this study had at least 10^7^ CFU/g, so it can be said that the growth of *L. acidophilus* is viable in all products obtained. In the salami-type sausages, it was observed that the mixture of microorganisms resulted in greater growth.

#### 3.3.1 Fermentation stage

*L. plantarum* and *L. acidophilus* are practical LAB that quickly dominate the microbial ecosystem during fermentation. LAB counts increase due to their ability to ferment sugars into lactic acid, lowering the meat mixture’s pH. Both LAB starting at ∼10^5^ CFU/g and increases to 10^6^ CFU/g by day 4. The ANOVA analysis revealed significant differences in microbial counts (log CFU/g) among the treatments with *L. plantarum*, *L. acidophilus*, and a mixture of both strains at various stages of fermentation and ripening. At the onset of fermentation (day 0), *L. acidophilus* exhibited significantly higher counts than *L. plantarum* and the mixture (*p* < 0.05). By the end of fermentation, *L. acidophilus* and the mixture showed similar, significantly elevated counts compared to *L. plantarum* (*p* < 0.05). This growth creates a competitive environment that inhibits the growth of spoilage microorganisms and pathogens (Leroy and De Vuyst, 2016). Moreover, the combination (starter + probiotic) may enhance the probiotic effect and slightly improve pathogen inhibition due to their complementary metabolic pathways (Hammes and Hertel, 2006).

#### 3.3.2 Ripening stage

The starter, probiotic, and mixture cultures grew during ripening by increasing between two and three log(CFU/g), reaching counts of 10^8^ and 10^9^ CFU/g after 15 days. During ripening, distinct patterns are observed: on day 6, the mixture displayed significantly higher counts than both *L. acidophilus* and *L. plantarum* (*p* < 0.05), and this trend continued through day 11, where the mixture reached the highest microbial count among all treatments (*p* < 0.05). At the end of ripening (day 15), the mixture maintained significantly higher microbial growth (*p* < 0.05). The Tukey pairwise comparisons confirmed that the mixed-culture treatment supported a more robust and sustained microbial proliferation throughout ripening, suggesting enhanced viability and metabolic activity under combined culture conditions. These findings underscore the statistical significance of using a mixed culture to achieve higher microbial counts during ripening, likely impacting the final characteristics of the salami-type product. In contrast, Hammes and Hertel (2006) reported LAB populations stabilize or slightly decrease (from 10^9^–10^10^ CFU/g to 10^8^ CFU/g by day 15) during ripening as nutrients deplete and moisture is reduced.

During salami production, microorganisms naturally present on raw materials or added as starters are responsible for fermentation, which must be carefully monitored for hygienic safety and quality. Studies aim to improve the survival of starter cultures and standardize the final product’s safety and quality (Blaiotta et al., 2018). In the production of cured/aged meat products like salami, the choice of starter cultures significantly impacts fermentation, flavor development, and functional properties. Each microorganism contributes to various aspects of the process when using *L. plantarum* as a starter, adding *L. acidophilus* as a probiotic, or employing a mixture of both. Recently, efforts have been made to develop meat-based functional foods by increasing beneficial compounds. Employing specific starter cultures aims to improve food safety and reduce costs. Functional foods with probiotic cultures, like LAB or bifidobacteria, can positively affect consumers’ health by maintaining intestinal microbial balance when consumed adequately (Blaiotta et al., 2018; Wang Y. et al., 2022).

*L. plantarum* is widely used in meat fermentation due to its acidifying capability to lower the pH of the meat product, providing safety (Leroy and De Vuyst, 2004). *L. acidophilus*, commonly used as a probiotic, can enhance the nutritional and health benefits of fermented meat products, is well-known for promoting gut health, and its incorporation into fermented meats allows the final product to offer potential probiotic effects (Huang and Adams, 2004; Holck et al., 2017). Probiotic survival through the fermentation and ripening processes can be challenging, but *L. acidophilus* has shown resistance when exposed to the acidic environment of fermented meat. Ensuring its survival is essential to delivering functional probiotic effects to consumers (Leroy and De Vuyst, 2004; Leeuwendaal et al., 2022). *L. acidophilus* contributes to acidification and can complement other functional properties; it may influence proteolytic and lipolytic activities, contributing to flavor and texture (Cocolin et al., 2009; Tran et al., 2011). Combining *L. plantarum* and *L. acidophilus* can create a more balanced fermentation process. Together, they may enhance flavor complexity and texture through combined proteolytic activity. The presence of *L. plantarum* may create a more favorable environment for *L. acidophilus* by rapidly lowering pH, which can potentially increase the survival of the probiotic strain during the fermentation process (Leroy and De Vuyst, 2016). When combining cultures, it is essential to monitor fermentation kinetics, as *L. plantarum* may outcompete *L. acidophilus* in terms of growth rate. Optimal formulation and fermentation conditions should be maintained to ensure that both microorganisms contribute to the final product (Hammes and Hertel, 2006). Other studies also demonstrated the great viability of starters and probiotics during fermentation and ripening of fermented meat products when using *Lactiplantibacillus plantarum* BFL probiotic strain to produce dry-fermented sausages (Sirini et al., 2023). They found the probiotic maintained high viability during drying and exhibited strong biocontrol effects.

During fermentation and ripening, *L. plantarum* and *L. acidophilus* can hydrolyze meat proteins into smaller peptides that exhibit antioxidant properties, helping to scavenge free radicals and reduce oxidative damage. This is particularly relevant in cured meat products, where lipid oxidation can negatively affect flavor, texture, and shelf life. Studies have demonstrated that peptides produced by *L. plantarum* can significantly enhance the antioxidant capacity of fermented meat products (Luan et al., 2021). Additionally, *L. acidophilus* has been shown to generate antioxidant peptides, further contributing to the product’s stability (Sohaib et al., 2017). Bioactive peptides derived from proteins during fermentation may exhibit antihypertensive effects by inhibiting angiotensin-I converting enzyme (ACE), which plays a crucial role in blood pressure regulation. *L. plantarum* and *L. acidophilus* have been shown to release ACE-inhibitory peptides in various food matrices, including fermented meat products (Toldrá et al., 2020). *L. acidophilus* LA-5 could provide health benefits via salami-type and delays gastrointestinal disorders by regulating the immune system, enhances lactose tolerance, regulates the intestinal microbiota, produces metabolites (lactic acid) that inhibit the growth of pathogenic bacteria, and improves the anti-inflammatory effects by modulating TLR expression (Anjum et al., 2014; Remes-Troche et al., 2020; Liu et al., 2024). Moreover, previous studies about probiotic sausages demonstrated that 30–50 g of daily intake are enough to obtain health benefits (Jahreis et al., 2002; Pérez-Burillo et al., 2020). However, further investigation is needed to clearly identify the health benefits of the salami-type containing *L. acidophilus* LA-5 formulated in the present study.

### 3.4 Microbial growth of contaminated microbiota during fermentation and ripening

Table 3 presents the changes in microbial counts of contaminating initial microbiota during the fermentation and ripening stages for the meat product inoculated with *L. plantarum* and *L. acidophilus* mixture. Initial counts were near ≤10^2^ CFU/g for *Staph. aureus*, total coliforms, *E. coli*, and yeasts and molds in fresh salami-type sausage, whereas *Salmonella* spp. and *L. monocytogenes* were under the detection limit (<10 CFU/g). Enterobacteriaceae is sensitive to acidification and becomes non-detectable by day 5–6. TMAB initial counts were ∼10^5^ CFU/g, which is slightly higher than those reported by Gambi et al. (2023) (4.04 log CFU/g) in Italian salami. Likewise, a decreasing behavior was observed in Enterobacteriaceae (∼4 log CFU/g) in Italian salami during ripening to close the detection limit (Lorenzo et al., 2018; Gambi et al., 2023); moreover, *Salmonella* spp. and *L. monocytogenes* were not detected (Gambi et al., 2023). *Staph. aureus*, and yeasts and molds are inhibited during the ripening. *Staph. aureus* is more resistant to pH changes but is inhibited by high LAB counts and due to the reduced moisture during ripening. A similar trend was obtained for *Staph. aureus* counts <10 CFU/g at the end of the ripening process (Maksimovic et al., 2018). These trends align with studies showing the efficacy of LAB in suppressing spoilage microorganisms and pathogens during the fermentation and ripening of cured meats (Leroy and De Vuyst, 2016; Ibrahim et al., 2021; Favaro and Todorov, 2017; Lorenzo et al., 2018; Pateiro et al., 2015). Probiotic cultures (*L. rhamnosus* GG and *L. plantarum* 299v) declined Enterobacteriaceae counts from 6.43 log CFU/g to under detection limit in Spanish type fermented sausages when microbial counts were compared with sausages without probiotics (Rubio et al., 2013a). In another study, the growth inhibition of enterobacteria in sturgeon sausages incorporating eight probiotics was observed (Wang et al., 2015). Thus, probiotic cultures contribute to enhancing the safety of fermented meats.

Bioactive peptides produced by LAB, especially *L. plantarum* and *L. acidophilus*, can also exhibit antimicrobial properties. These peptides, known as bacteriocins, can inhibit the growth of spoilage microorganisms and pathogens, including *L. monocytogenes*, *Staph. aureus*, and *Salmonella* spp. (An et al., 2017; Wang D. et al., 2022). For example, *L. plantarum* has been shown to produce plantaricins, which exhibit potent antimicrobial effects and could help reduce the need for synthetic preservatives in meat products (Gänzle, 2015). Combining *L. plantarum* and *L. acidophilus* could further enhance this antimicrobial effect, potentially improving food safety. This is particularly important in meeting the strict microbiological standards for products like salami. For instance, S*almonella* must have an initial count of 0 CFU/g, as any presence is unacceptable in ready-to-eat products (FDA, 2023). Similarly, *L. monocytogenes*, with an initial count of 0 CFU/g in the final product and <1 CFU/g in processing areas, *Staph. aureus* should remain below 10^2^ CFU/g to minimize toxin production (EFSA Panel on Biological Hazards, 2020). Additionally, microbial quality indicators like total viable count should start at 10^3^–10^5^ CFU/g and decrease during fermentation and drying (Rocchetti et al., 2021), while Enterobacteriaceae counts should remain at or below 10^2^ CFU/g, indicating proper sanitary practices (ISO, 2017). Therefore, based on these criteria, the salami-type sausages of this study fulfill the microbial limits established by the regulatory agencies.

### 3.5 Sensory analysis

**FIGURE 3:**
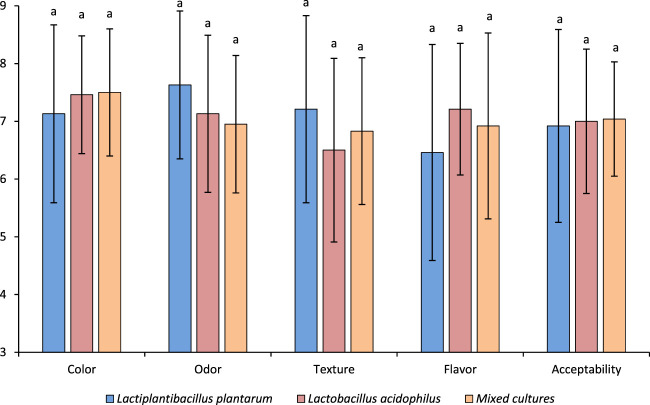
Average scores of salami-type products’ sensory evaluation formulated with starter culture (*Lactiplantibacillus plantarum*), probiotic (*Lactobacillus acidophilus*), and the mixture of both.

Figure 3 shows the sensory evaluation scores for salami-type sausages fermented with *L. plantarum, L. acidophilus*, and mixed culture. The average scores were compared across color, odor, texture, flavor, and overall acceptability. The ANOVA showed no significant differences (*p* > 0.05) in these attributes, indicating that the type of lactic acid bacteria did not noticeably affect sensory qualities. While the mixed culture did not achieve higher ratings, it may introduce slight notes that may enhance consumer preference. Likewise, small differences in sensory attributes (redness, raw odor and acidic taste) between control and probiotic dry-fermented pork sausages were previously reported (Pavli et al., 2020). *Fuets* (a Spanish type of small caliber low-acid fermented sausages) with different probiotics (*L. plantarum* 299v, *L. casei* Shirota, and *L. rhamnosus* GG) had similar (*p* > 0.05) sensory quality on appearance, odor, flavor, oral texture, and overall acceptability compared with *Lactobacillus* starters (Rubio et al., 2013b). Lactic acid fermentation influences the flavor profile by producing organic acids and contributing to proteolysis and lipolysis, which release flavor precursors such as fatty acids, peptides, amino acids, and aldehydes (Hammes and Hertel, 2006; Berardo et al., 2017; Wang Y. et al., 2022). Also, *L. plantarum* contributes to protein degradation during ripening, enhancing texture by forming peptides that improve water-binding capacity and reduce product hardness (Cao et al., 2019; Leroy and De Vuyst, 2004). In other study, probiotic salamis (*L. rhamnosus* LR32 200B or *L. plantarum* LP115 400B) were more liked than control sample in color and flavor, indicating that probiotics contributes to health benefits and sensory quality of salami (Tukel and Sengun, 2024). The sensory properties (color, consumer acceptability, hardness and springiness) of sturgeon sausages were improved when eight probiotics were included in the formulation/fermentation (Wang et al., 2015).

The mean color scores were similar across all treatments, suggesting that lactobacilli strains do not alter the color development of the salami-type meat (Figure 3). This is consistent with findings by Chen et al. (2016), who reported that *L. plantarum* strains were able to maintain color stability in fermented sausages, as these bacteria often contribute to nitrite reduction, enhancing the typical reddish-pink color of cured meats through myoglobin nitrosylation. Similarly, Ba et al. (2018) demonstrated that *L. plantarum* can induce nitrite reductase activity, which plays a critical role in maintaining the characteristic color of cured meats. The odor scores varied slightly between treatments, with *L. plantarum* receiving higher mean scores than mixed culture or *L. acidophilus* alone. The production of volatile compounds such as lactic acid, aldehydes, and alcohols during fermentation by lactobacilli contributes to the aroma of fermented products. Previous studies have shown that different lactobacilli strains produce different profiles of volatile compounds, but the impact on consumer perception of odor is often subtle (Bis-Souza et al., 2019; Ojha et al., 2015). The lack of significant differences in odor among the treatments in this study indicates that these strains, even when mixed, do not dramatically alter the aroma profile.

The salami-type samples’ textural properties were not significantly different, with *L. plantarum* showing slightly higher scores. During fermentation, pH, water retention capacity, and protein denaturation often affect texture. Research has shown that *L. plantarum* strains can contribute to more efficient protein hydrolysis, potentially enhancing the firmness of the product (Ashaolu et al., 2023). However, the similarities in texture scores in this study suggest that all three treatments maintained consistent structural integrity and mouthfeel. Flavor is a key determinant of acceptability, and while *L. acidophilus* scored slightly higher; the differences were not statistically significant (*p* > 0.05). The production of organic acids, peptides, and fatty acid derivatives during fermentation influences the flavor of fermented products. The similar flavor scores suggest neither strain nor their combination led to off-flavors or undesirable sensory deviations. The overall acceptability scores did not show significant differences (*p* > 0.05), suggesting that probiotic *Lactobacillus* does not negatively impact the sensory appeal of salami-type products. This agrees with Sirini et al. (2023), who found that probiotic strains can be successfully incorporated into fermented sausages without compromising consumer acceptability. Probiotics’ ability to survive fermentation while maintaining acceptable sensory qualities makes them viable for producing functional meat products with added health benefits.

The results suggest practical implications for producing and marketing probiotic-enriched fermented meat products. Although sensory attributes showed no significant differences among treatments, incorporating *L. acidophilus* and *L. plantarum* can enhance their functional value. This aligns with increasing consumer demand for gut health foods, enabling producers to market these salami-type products with added health benefits (Munekata et al., 2022; Carneiro et al., 2024). Incorporating probiotics into fermented meats allows differentiation in a competitive market, appealing to health-conscious consumers (Dey, 2018). These products’ proven sensory appeal supports their potential for broader acceptance in typical markets.

### 3.6 Conclusion

The salami-type meat product showed good growth of starter and probiotic microorganisms, with populations between 10^8^ and 10^9^ CFU/g. Declining pH and increasing titratable acidity were obtained during fermentation and ripening of the salami-type sausages for 15 days without differences between starter and probiotic or the mixture of cultures. The sensory analysis revealed that the type of bacteria used did not affect the product’s characteristics. The findings of this study suggest that it is feasible to produce a probiotic salami-type sausage using *L. acidophilus* or a mixture with a starter without affecting the sensory characteristics. The present findings provide information for potential applications at the industrial level to manufacture probiotic salami-type products. The inclusion of probiotics not only improves the product’s health benefits but also enhances the product’s safety. Despite individual inoculation results indicating that the formulation is suitable for their growth, further analysis is needed to identify if any microorganisms (*L. acidophilus* or *L. plantarum*) predominate in the mixture. It may also be helpful to analyze volatile compounds and acetic acid produced during fermentation and ripening and the potential production of bacteriocins or bioactive peptides during these stages. In addition, health benefits for the intake of this probiotic salami-type could be investigated as well as the capability of the starter and probiotic to survive at gastrointestinal conditions.

## Data Availability

The raw data supporting the conclusions of this article will be made available by the authors, without undue reservation.
